# Detection of Live Shiga Toxin-Producing *Escherichia coli* with Long-Read Sequencing

**DOI:** 10.3390/ijms26052228

**Published:** 2025-03-01

**Authors:** Katrina L. Counihan, Shannon Tilman, Chin-Yi Chen, Yiping He

**Affiliations:** Eastern Regional Research Center, Agricultural Research Service, United States Department of Agriculture, Wyndmoor, PA 19038, USA; shannon.tilman@usda.gov (S.T.); chin-yi.chen@usda.gov (C.-Y.C.); yiping.he@usda.gov (Y.H.)

**Keywords:** propidium monoazide, ethidium monoazide, foodborne pathogens, method development, long-read sequencing

## Abstract

A requirement of any foodborne pathogen testing method is that it only detects live bacteria. Ethidium monoazide (EMA) and propidium monoazide (PMA) are dyes that penetrate the membranes of dead cells and form cross-linkages in the DNA, which prevents its amplification in PCR. This study investigated whether treatment with EMA or PMA would inhibit the sequencing of DNA from dead *Escherichia coli*. Range finding experiments with qPCR were conducted to determine the optimal concentrations of EMA and PMA needed to inhibit the amplification of DNA from dead cells while not influencing live cells. An EMA concentration that differentiated between live and dead cells could not be established. However, a PMA concentration of 25 µM effectively prevented qPCR amplification of DNA from dead *E. coli* while not impacting the amplification of live *E. coli* DNA. Sequencing experiments were conducted with PMA-treated live, untreated live, PMA-treated dead, and untreated dead *E. coli*. There were no significant differences in the detection of virulence genes of interest between the PMA-treated live, untreated live, and untreated dead *E. coli*. However, no DNA sequencing data were obtained from the PMA-treated dead *E. coli*. These results suggest that PMA could be incorporated into sample preparation methods prior to sequencing to selectively detect live cells of foodborne pathogens.

## 1. Introduction

Foodborne illnesses remain common in the United States (U.S.), with an estimated 48 million people getting sick annually [1]. Shiga toxin-producing *Escherichia coli* (STEC) is a foodborne pathogen that generally causes diarrhea and vomiting, but severe disease, such as hemorrhagic colitis or hemolytic uremic syndrome, may occur [2]. The incidence rate of STEC infection is 5.7 per 100,000 people, and has been increasing since 2018 [3]. Pathogen contamination in food can also cause significant economic losses. Over 15 million pounds of meat were recalled in the U.S. during 2021, and two of those recalls were due to STEC contamination [4]. It is estimated that foodborne pathogen contamination costs the U.S. economy approximately USD 17 billion annually [5].

The U.S. Department of Agriculture Food Safety and Inspection Service (USDA FSIS) isolates and identifies STEC as an adulterant in meat through a combination of culturing, molecular methods, O-antigen typing, and matrix-assisted laser desorption/ionization time-of-flight (MALDI-TOF) mass spectrometry [6]. A food sample that tests positive for the *stx* and *eae* genes and one of seven targeted O-antigens commonly associated with STECs isolated from symptomatic patients is considered adulterated [6]. The *eae* gene encodes intimin, which mediates enterocyte colonization, and expression of the *stx* gene produces Shiga toxin, which causes cytotoxicity in the host [2]. The STEC serotype O157:H7 is most frequently associated with foodborne outbreaks [3]. The *E. coli* serotype is determined by the O-antigen, which is present on the outer membrane, and the H-antigen, encoded by *fliC*, which is present on the flagella [7]. The ribosomal 16S rRNA gene, *rrsC*, is also targeted with RT-PCR for species identification [8]. At least four days are required to complete the current culture-based identification process [6]. Novel methods using long-read, whole-genome sequencing could reduce the amount of time needed for foodborne pathogen identification.

The MinION sequencer produced by Oxford Nanopore Technologies sequences RNA or DNA by detecting specific changes to the electrical current as strands pass through nanopores on a flow cell [9]. Long reads are generated that facilitate genome assembly [10], and real-time analysis allows pathogen detection to be accomplished in hours instead of days [11]. Whole-genome analysis can be conducted outside of traditional laboratories because the sequencers are small and portable, and the cost is generally lower than second-generation sequencing. The raw read accuracy of nanopore sequencing has also improved to >99.9% [12].

A disadvantage of DNA-based detection methods, such as PCR and sequencing, is that DNA from both live and dead organisms can be detected, but only living pathogens can infect and cause disease. Extracellular DNA and DNA in dead cells can persist for days to weeks [13,14]. Detecting DNA from dead bacteria could overestimate pathogen abundance [15] and potentially result in costly and unnecessary food recalls [16]. Therefore, culture-based methods remain the gold standard, because bacterial growth indicates viability [17]. For sequencing to become a practical food testing method, it will need to differentiate between live and dead pathogens.

Ethidium monoazide (EMA) and propidium monoazide (PMA) are derivatives of ethidium bromide and propidium iodide, respectively, with the addition of an azide group that allows the chemicals to bind to DNA covalently upon photoactivation [18,19]. PMA also differs from EMA in that it has two positive charges as opposed to one [18]. Both EMA and PMA intercalate double-stranded DNA that is extracellular, or by penetrating the compromised membranes of dead cells [18,20]. EMA and PMA have been used to prevent the amplification of DNA from dead cells in PCR and qPCR [15,21,22,23]. Both EMA and PMA have also been used to prevent the sequencing of dead bacteria, but detection was limited to genus or species, not serotype [24,25,26]. PMAxx is a proprietary version of PMA that is more effective at discriminating between live and dead cells [27]. Therefore, the goal of this project was to determine if EMA or PMAxx could prevent the sequencing of DNA from dead STEC, but not interfere with the detection of serotype-specific genes of interest in live cells.

## 2. Results and Discussion

Triplicate tubes of heat-treated *E. coli* were plated in each experiment, and no growth was observed, which confirmed that the bacteria were killed at 90 °C for 10 min. Effective heat killing occurred starting after 5 min of incubation at 90 °C in a range finding experiment. The 10 min time point was chosen as it had been used in previous experiments in our lab, and to ensure that the *E. coli* were dead and not in a state of being viable but not culturable. Plating also determined that the *E. coli* concentrations in the EMA and PMAxx range finding experiments were 2 × 10^8^ cfu mL^−1^. Triplicate samples of live PMAxx-treated and untreated *E. coli* were also plated in the PMAxx sequencing experiment. These samples were plated to confirm the inoculum concentration and to ensure the PMAxx treatment did not impact the viability or culturability of the *E. coli*. Any lethality due to PMAxx would result in false negatives and make a testing method incorporating it unacceptable for regulatory testing. A range of 3.2–4 × 10^8^ cfu mL^−1^ was observed in the plating results, which substantiated the *E. coli* inoculum concentration and indicated the 25 µM PMAxx was not lethal to the bacteria.

**Figure 1:**
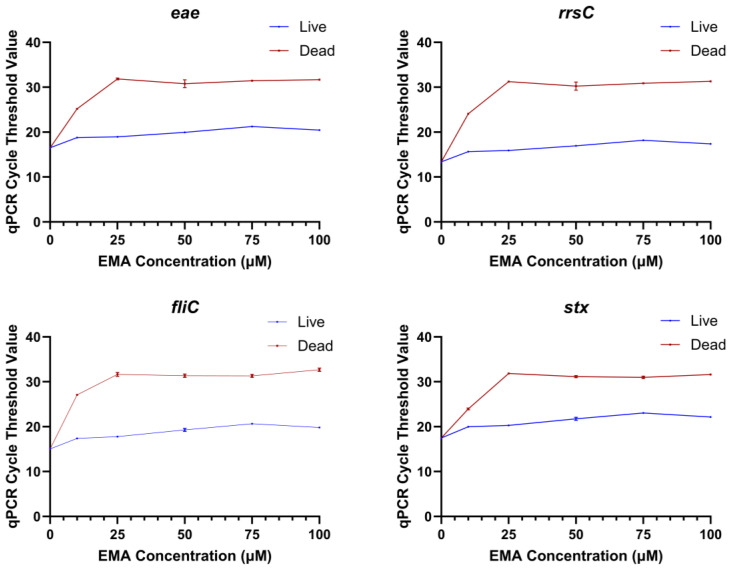
qPCR cycle threshold values for amplification of the genes *eae*, *rrsC*, *fliC*, and *stx* in DNA extracted from live or dead *Escherichia coli* treated with different concentrations of EMA in the range finding experiment.

**Figure 2:**
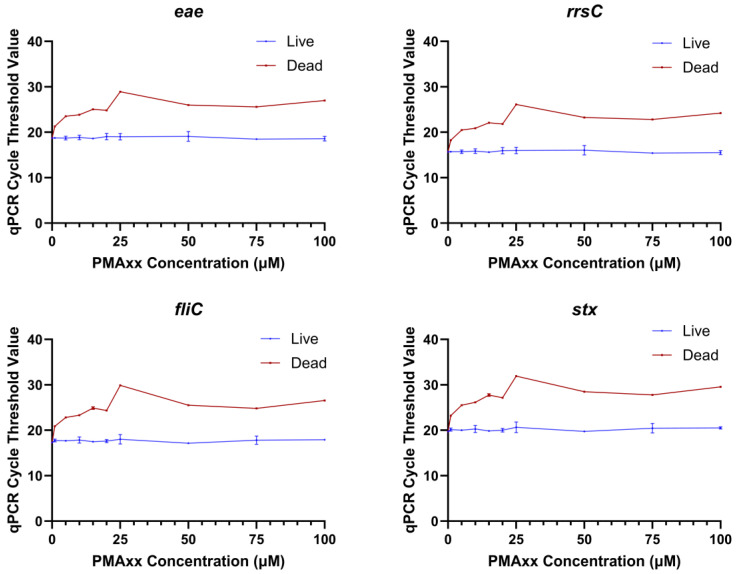
qPCR cycle threshold values for amplification of the genes *eae*, *rrsC*, *fliC*, and *stx* in DNA extracted from live or dead *Escherichia coli* treated with different concentrations of PMAxx in the range finding experiment.

A range of EMA and PMAxx concentrations were tested to determine the optimal concentration of each chemical that would prevent the detection of dead bacteria while not affecting the detection of live bacteria. The range finding experiments were conducted with qPCR because numerous studies have shown that EMA and PMA can block the amplification of dead cells [15,21,22,23]. The Ct values of dead STEC treated with 10, 25, 50, 75, or 100 µM EMA were significantly higher than the dead untreated control (0 µM EMA) at all concentrations tested (Figure 1). However, the Ct values of EMA-treated live bacteria were also significantly higher as compared to the untreated live control (0 µM EMA) at all concentrations. This indicated that an EMA concentration lower than 10 µM would be needed for live bacterial detection to not be impacted. However, the Ct values for dead STEC tested with 10 µM EMA were closer to the Ct values for EMA-treated live bacteria, especially for the *stx* gene. These results suggested that a lower treatment concentration of EMA would not be able to differentiate between live and dead bacteria. A higher risk of false positives or false negatives would not be acceptable for regulatory testing. Therefore, no further experiments were conducted with EMA. In the PMAxx range finding experiments, there were no statistical differences in qPCR Ct values between PMAxx-treated live cells and the untreated live control. Dead PMAxx-treated cells had significantly higher Ct values than the live untreated control and live *E. coli* treated with the same concentration of PMAxx (Figure 2). The Ct values of PMAxx-treated dead STEC increased with PMAxx concentration until 25 µM, and then the values stabilized. The 25 µM PMAxx concentration was selected for the sequencing experiments as it provided the maximum inhibition of amplification of dead cells in qPCR while not influencing live cell amplification.

**Figure 3:**
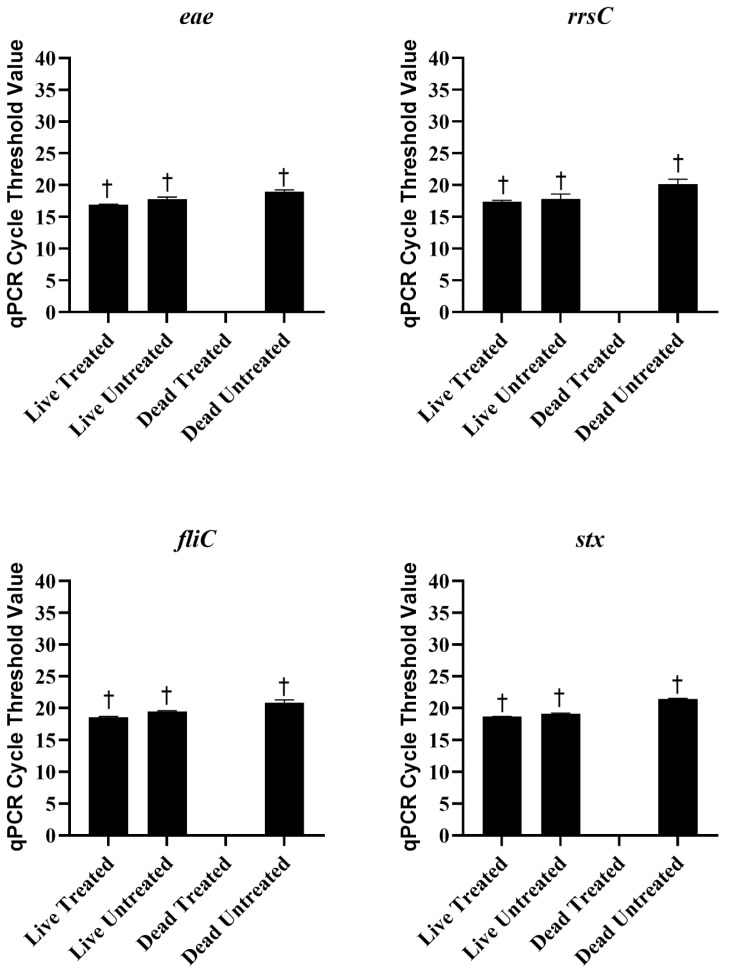
Mean ± standard error of cycle threshold values for live and dead *Escherichia coli* treated or untreated with 25 µM of PMAxx in the qPCR assay to detect the genes of interest, *eae*, *rrsC*, *fliC*, and *stx*. The cycle threshold values for dead treated samples were so low that the software returned an undetermined result. † indicates a significantly different Ct value than the dead treated sample.

**Figure 4:**
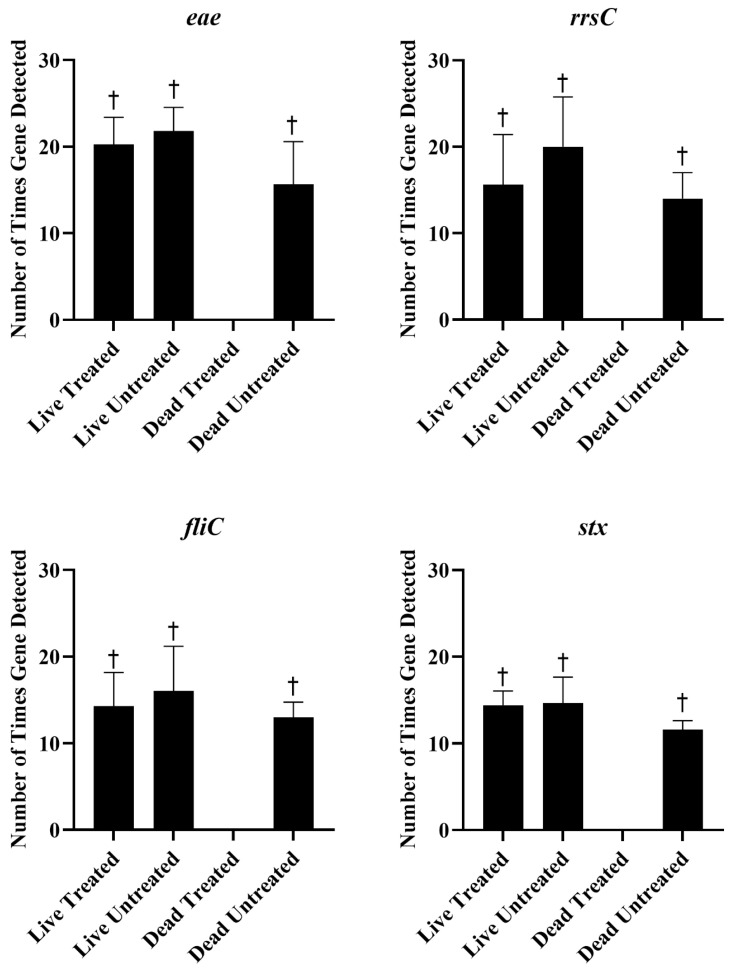
The mean ± standard error of the number of times each of the genes of interest, *eae*, *rrsC*, *fliC*, and *stx*, were detected using long-read sequencing of live and dead *Escherichia coli* treated or untreated with 25 µM of PMAxx. † indicates significantly higher detection than the dead treated sample.

Live and heat-killed STEC were either treated with 25 µM PMAxx or left untreated. The DNA was extracted from each sample and tested with qPCR and sequencing. The statistics of the sequencing run are provided in Table 1. There were no significant differences in Ct values between live untreated, live PMAxx-treated, or dead untreated *E. coli* (Figure 3). However, no amplification was observed in dead PMAxx-treated *E. coli*, which was significantly different from the other treatments (Figure 3). The concentration of DNA extracted from the live cells was significantly higher than the concentration extracted from the dead cells, regardless of PMAxx treatment (Table 2). Nucleases in the dead cells may have degraded DNA and resulted in less being extracted [28]. The amount of DNA extracted from PMAxx-treated dead cells was also lower as compared to untreated dead cells, although not significantly. In addition to inhibiting PCR amplification, the covalent binding of PMAxx to DNA is also supposed to hinder its purification during extraction procedures [27]. The combination of DNA degradation and PMAxx binding resulted in very low concentrations of DNA being extracted from the treated dead cells. However, despite the lower concentration of DNA extracted from the dead untreated cells, detection of the genes of interest was not significantly different as compared to the treated or untreated live *E. coli*, and the virulence genes, *fliC*, *eae*, and *stx*, and the 16S rRNA gene, *rrsC*, were all detected around the same frequency (Figure 4). There were no significant differences in the detection of the genes of interest between treated and untreated live *E. coli*, indicating that PMAxx did not penetrate live cells to prevent DNA sequencing. However, the genes were not detected in PMAxx-treated dead *E. coli*, and this was significant as compared to untreated dead *E. coli* and treated or untreated live *E. coli*. Together, these findings suggest that PMAxx penetrated dead cells and intercalated the DNA to effectively inhibit sequencing.

Testing meat products for foodborne pathogen contamination is important to prevent illness in people consuming these products. Previous studies have shown that long-read sequencing has potential as a method for foodborne pathogen detection [29]. However, a major weakness of sequencing is that any DNA in a sample, whether from a live or dead cell, will be sequenced. For the purposes of food safety testing, only live bacteria are of concern because they are able to infect and cause disease [30]. Many food manufacturing practices include intervention steps to inactivate microbes that are pathogenic or cause spoilage [30]. Bacteria that have been killed through intervention would not be detected with culture-based methods, but they may be with sequencing methods, which could lead to unnecessary remediation measures. Therefore, procedures need to be developed for sequencing methods that result in the differentiation between live and dead bacteria.

The chemicals EMA and PMAxx can penetrate the membrane of dead cells and form cross-linkages with DNA when photoactivated, which then inhibits amplification in PCR [23,31]. It was hypothesized that cross-linking EMA or PMAxx to the DNA of dead bacteria would also prevent sequencing. Long-read sequencing was conducted with the Oxford Nanopore Technologies’ MinION, which functions by separating double-stranded DNA so that a single strand passes through a nanopore, and each base disrupts the electrical current to produce a distinct pattern that can be basecalled by software [9]. If the DNA strand is unable to separate, sequencing will not occur, and this was observed with both chemicals.

The current methods used by USDA FSIS to identify the most common bacterial foodborne pathogens, STEC, *Salmonella*, and *Campylobacter*, rely on selective culturing and molecular diagnostic tests, and take at least four days to obtain a positive confirmation [6,32,33]. Antimicrobial susceptibility testing and whole-genome sequencing are employed to further characterize *Salmonella* and *Campylobacter* isolates [32,33]. The results of this study suggest that methods could be developed to incorporate PMAxx treatment during sample preparation to prevent the sequencing of dead bacteria. This would overcome a major disadvantage of sequencing in that only live bacteria would be detected. It may also enhance detection sensitivity because the DNA from dead bacteria would compete less for available sequencing pores, increasing the likelihood that DNA from live pathogens get sequenced. Additionally, eliminating growth in culture to isolate live pathogens would significantly shorten testing time. Sequencing would also be advantageous because it would replace molecular diagnostic tests, saving additional time and money. For example, during STEC testing, there are one MALDI and three PCR assays that could be eliminated. Sequencing would identify all targets of the molecular assays and provide data on antimicrobial resistance genes and serotype. However, there are also challenges that will need to be addressed. This study sequenced PMAxx-treated *E. coli* from pure cultures as an initial proof of concept. The presence of a food matrix may require higher concentrations of PMAxx, or the need to separate the pathogen from the food matrix prior to treatment. Also, PMAxx may penetrate bacterial cells differently, especially Gram-positive bacteria that have different cell wall compositions and which do not possess lipopolysaccharide. The concentration may need to be refined if multiple species are to be targeted.

Despite these potential challenges, this study demonstrated the ability of PMAxx to effectively prevent the sequencing of dead *E. coli*. Sequencing-based detection of only live bacteria could be accomplished in one to two days, which would cut testing time in half compared to current methods. Multiple live pathogens could also be detected in one sequencing run while providing additional information on antibiotic-resistance genes, virulence genes, and serotypes. Further research is warranted to investigate the use of PMAxx in different sample preparation methods prior to sequencing to fully elucidate its practicality.

## 3. Materials and Methods

### 3.1. Bacterial Culturing

*Escherichia coli* O157:H7 (ATCC 43895) was grown in modified tryptic soy broth (mTSB) overnight at 42 ± 1 °C at 100 rpm. A temperature of 42 °C was used because this is the temperature at which USDA FSIS incubates sample enrichments for *E. coli* testing [6]. The bacterial concentration was determined by measuring the optical density at a wavelength of 600 nm (OD 600) with a Denovix DS-11 FX+ spectrophotometer (DeNovix Inc., Wilmington, DE, USA). Serial dilutions were plated on Luria–Bertani agar (LB; (Thermo Fisher Scientific Chemicals, Inc., Ward Hill, MA, USA)) to confirm cell concentrations (cfu mL^−1^).

### 3.2. EMA and PMAxx Range Finding Experiments

Clear 1.5 mL microcentrifuge tubes were prepared with 0.4 mL of 1 × 10^8^ cfu mL^−1^ *E. coli* culture. The bacteria in half of the tubes were heat killed by incubating them at 90 °C for 10 min. The amount of time needed to heat kill *E. coli* had been determined by incubating 1 mL aliquots of the bacteria at 90 °C and removing samples every minute for a total of 10 min for culturing. The contents of 1 of the heat killed tubes was plated on LB agar to confirm there was no growth. The other tubes were left alive. For the EMA range finder, each of the following concentrations of ethidium monoazide (EMA, Biotium Inc., Fremont, CA, USA) were added to 1 live tube and 1 dead tube, except for the 100 µM treatment, which was added to 2 live tubes and 1 dead tube: 10 µM, 25 µM, 50 µM, 75 µM, and 100 µM. A live control had no EMA added to it. All of the tubes were incubated at room temperature in a thermomixer with 100 rpm rotation for 5 min in the dark. For the PMAxx range finder, all tubes, except 1 live control, had 100 µL of 5X Propidium Monoazide (PMA) Enhancer for Gram-negative Bacteria (Biotium Inc.) added. Next, each of the following concentrations of PMAxx (Biotium Inc.) were added to 1 live tube and 1 dead tube, except for the 100 µM treatment, which was added to 2 live tubes and 1 dead tube: 1 µM, 5 µM, 10 µM, 15 µM, 20 µM, 25 µM, 50 µM, 75 µM, and 100 µM. No PMAxx was added to the control. All tubes were incubated at room temperature in a thermomixer with 100 rpm rotation for 25 min in the dark. The tubes were then placed in a PMA-Lite LED Photolysis Device (Biotium Inc.) for 15 min. The tubes of live bacteria from the 100 µM EMA and PMAxx treatments were serially diluted and plated on LB agar to confirm that the EMA and PMAxx did not cause mortality. The remaining tubes were centrifuged at 5000× *g* for 10 min and the supernatant was removed. The bacteria in all tubes were washed with 1 mL phosphate-buffered saline (PBS) and then centrifuged again at 5000× *g* for 10 min. After that, the supernatant was removed and the DNA was extracted from the pellet using the NucleoSpin^®^ Microbial DNA kit (Macherey-Nagel, Allentown, PA, USA) according to the manufacturer’s instructions.

### 3.3. Optimized PMAxx Experiment

An experiment was conducted with 4 treatments consisting of 6 replicates: live PMAxx-treated *E. coli*, dead PMAxx-treated *E. coli*, untreated live *E. coli*, and untreated dead *E. coli*. The samples were prepared as described above for the PMAxx range finding experiment. PMAxx-treated samples had 25 µM of PMAxx added, which was the optimal concentration determined in the range finding experiment. Three tubes from each of the treatments were plated on LB agar to confirm expected bacterial concentrations. The remaining tubes were used for DNA extractions.

### 3.4. Quantitative Polymerase Chain Reaction

Quantitative real-time polymerase chain reaction (qPCR) was used to test for the presence of the *fliC*, *stx*, *eae*, and *rrsC* genes of *E. coli* O157:H7 in the DNA extracted from the samples in the range finding and optimized experiments, following established USDA FSIS protocols [8]. A QuantStudio 5 Real-Time PCR System (Applied Biosystems, Waltham, MA, USA) was used with a TaqMan^TM^ Environmental Master Mix 2.0 (Applied Biosystems) for qPCR.

### 3.5. Long-Read Sequencing

Libraries were prepared using the DNA extractions from the samples in the optimized PMAxx experiment with the Rapid Barcoding Kit (SQK-RBK004, Oxford Nanopore Technologies [ONT], Oxford, UK) according to the manufacturer’s instructions. A flow cell check was performed prior to sequencing to ensure enough pores were available for sequencing. The prepared libraries were run for 24 h on a MinION Mk1B sequencer (ONT) with R9 flow cells (ONT).

### 3.6. Data Analysis

Sequencing data were basecalled in real time or post-run with MinKNOW software version 22.03.6 (ONT). The reads were filtered for quality in MinKNOW software, removing sequences with a quality score of less than 8 or a read length of less than 1 kb. The fastQ files that passed the quality check were imported into Geneious Prime software (version 2023, GraphPad Software, LLC, Boston, MA, USA) and aligned to an STEC reference genome (NCBI Accession #NC002695.5) using Minimap2 (version 2.24) [34]. The target genes, *fliC*, *stx*, *eae*, and *rrsC*, were searched for in the alignment, and the number of times each gene was detected was determined. The mean and standard error were calculated for triplicates. A *t*-test was performed to determine statistical differences (*p* < 0.05) between treatments.

## Figures and Tables

**Table 1:** Statistics for the sequencing run of the live and dead *Escherichia coli*, treated or untreated with PMAxx.

Sample	Mean Length ± Standard Error (b)	Minimum Length (b)	Maximum Length (b)	Reads Generated ± Standard Error (k)	Estimated Bases (Mb)	Data Produced (GB)	Estimated N50 (kb)
Live Untreated	3476 ± 74.31	1000	22,964	89,267 ± 8230	1552.00	19.31	4.19
Live Treated	3552 ± 74.31	1000	32,592	78,544 ± 22,504			
Dead Untreated	2510 ± 220.10	1000	20,560	69,560 ± 8619			
Dead Treated	3049 ± 95.35	1000	9947	10.67 ± 5.36			

**Table 2:** Concentration of DNA extracted from live and dead *Escherichia coli*, treated or untreated with PMAxx.

Sample	Concentration (ng µL^−1^)
Live Untreated *	33.24 ± 1.75
Live Treated *	34.82 ± 1.79
Dead Untreated	8.85 ± 1.87
Dead Treated	2.90 ± 0.22

* DNA concentration was significantly higher than the dead untreated and treated cells.

## Data Availability

The raw data supporting the conclusions of this article are available at https://doi.org/10.15482/USDA.ADC/28317536 [35].

## Abbreviations

The following abbreviations are used in this manuscript:

EMA	Ethidium monoazide
PMA	Propidium monoazide
US	United States
STEC	Shiga toxin-producing Escherichia coli
USDA FSIS	United States Department of Agriculture Food Safety and Inspection Service
MALTI-TOF	Matrix-assisted laser desorption/ionization time-of-flight
mTSB	Modified tryptic soy broth
LB	Luria–Bertani
OD	Optical density
cfu	Colony-forming unit
mL	Milliliter
PBS	Phosphate-buffered saline
qPCR	Quantitative real-time polymerase chain reaction
ONT	Oxford Nanopore Technologies
NCBI	National Center for Biotechnology Information
